# A systems biology approach to analyse leaf carbohydrate metabolism in *Arabidopsis thaliana*

**DOI:** 10.1186/1687-4153-2011-2

**Published:** 2011-06-17

**Authors:** Sebastian Henkel, Thomas Nägele, Imke Hörmiller, Thomas Sauter, Oliver Sawodny, Michael Ederer, Arnd G Heyer

**Affiliations:** 1 Institut für Systemdynamik, Universität Stuttgart, D-70550 Stuttgart, Germany; 2 Biologisches Institut, Abteilung Pflanzenbiotechnologie, Universität Stuttgart, Pfaffenwaldring 57, D-70550 Stuttgart, Germany; 3 Life Science Research Unit, Université du Luxembourg, L-1511 Luxembourg, Germany

**Keywords:** Systems biology, carbohydrate metabolism, *Arabidopsis thaliana*, kinetic modelling, stability analysis, sucrose cycling

## Abstract

Plant carbohydrate metabolism comprises numerous metabolite interconversions, some of which form cycles of metabolite degradation and re-synthesis and are thus referred to as futile cycles. In this study, we present a systems biology approach to analyse any possible regulatory principle that operates such futile cycles based on experimental data for sucrose (Scr) cycling in photosynthetically active leaves of the model plant *Arabidopsis thaliana*. Kinetic parameters of enzymatic steps in Scr cycling were identified by fitting model simulations to experimental data. A statistical analysis of the kinetic parameters and calculated flux rates allowed for estimation of the variability and supported the predictability of the model. A principal component analysis of the parameter results revealed the identifiability of the model parameters. We investigated the stability properties of Scr cycling and found that feedback inhibition of enzymes catalysing metabolite interconversions at different steps of the cycle have differential influence on stability. Applying this observation to futile cycling of Scr in leaf cells points to the enzyme hexokinase as an important regulator, while the step of Scr degradation by invertases appears subordinate.

## Introduction

Plant metabolic pathways are highly complex, comprising various branch points and crosslinks, and thus kinetic modelling turns up as an adequate tool to investigate regulatory principles. Recently, we presented a kinetic modelling approach to investigate core reactions of primary carbohydrate metabolism in photosynthetically active leaves of the model plant *Arabidopsis thaliana *[1] with an emphasis on the physiological role of vacuolar invertase, an enzyme that is involved in degradation of sucrose (Scr). This model was developed in an iterative process of modelling and validation. A final parameter set was identified allowing for simulation of the main carbohydrate fluxes and interpretation of the system behaviour over diurnal cycles. We found that Scr degradation by vacuolar invertase and re-synthesis involving phosphorylation of hexoses (Hex) allows the cell to balance deflections of metabolic homeostasis during light-dark cycles.

In this study, we investigate the structural and stability properties of a model derived from the Scr cycling part of the metabolic pathway described in [1]. Based on the existing model structure, model parameters were repeatedly adjusted in an automated process applying a parameter identification algorithm to match the measured and simulated data. A method for statistical evaluation of the parameters and simulation results is introduced, which allows for the estimation of parameter variability. Statistical evaluation demonstrates that the same nominal concentration courses are predicted for different identification runs, while small variability in fluxes and larger variability in parameters can be observed. Further, the parameter identification results were analysed applying a principal component analysis (PCA). This leads to a more extensive investigation with respect to the extension and alignment of the parameter values in the parameter space. In addition, this allows for conclusions concerning the identifiability of the parameters and the confirmation that the cost function is sensitive along parameter combinations. An investigation of structural stability properties of Scr cycling showed feedback inhibition of Hex on invertase and sugar phosphates (SP) on hexokinase likely to be involved in stabilisation of the metabolic pathway under consideration. Feedback inhibition of hexokinase was more efficient in stabilising Scr cycling than inhibition of invertase, indicating that, at this step of the cycle, a superior contribution to stabilisation of homeostasis can be achieved.

## The central carbohydrate metabolism in leaves of *A. thaliana*

**Figure 1:**
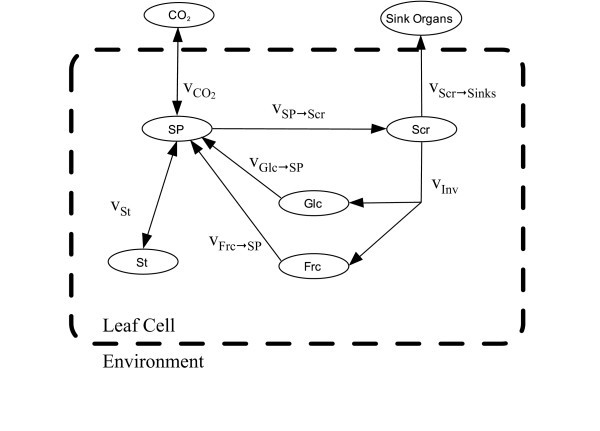
**Model structure of the central carbohydrate metabolism in leaves of *A. thaliana***. SP, sugar phosphates; St, starch; Scr, sucrose; Glc, glucose; Frc, fructose. *v *represent rates of metabolite interconversion.

Within a 24-h light/dark cycle, two principal modes of metabolism can be distinguished for plant leaves: photosynthesis (day), and respiration (night). During the day, carbon dioxide is taken up, and storage compounds like starch (St) accumulate, while this stock is in part respired during the night. Under normal conditions, a certain proportion of carbon is fixed as new plant biomass. However, typical source leaves as considered here are mature, and thus carbon use for growth can be neglected. Therefore, the carbon balance is completely determined by photosynthesis, respiration and carbon allocation to associated pathways or heterotrophic tissues that are not able to assimilate carbon on their own. Based on this information and known biochemical reactions, a simplified model structure for the interconversion of central metabolites was created (Figure 1).

The compounds SP, St, Scr, glucose (Glc) and fructose (Frc) are derived from photosynthetic carbon fixation and linked by interconverting reactions. The flux  represents the rate of net photosynthesis, i.e. the sum of photosynthesis and respiration. Carbon exchange with the environment and intracellular interconversions are linked through the pool of SP. This pool is predominantly constituted by the phosphorylated intermediates glucose-6-phosphate and fructose-6-phosphate. SP can reversibly be converted to St through the reaction *v*_St_. The reaction *v*_SP_→_Scr _represents a set of reactions leading to Scr synthesis. Among them, the reaction of Scr phosphate synthase is considered the rate-limiting step [2]. Scr can either be exported, for example, by a transport to sinks *v*_SP_→_Sinks_, or cleaved into Glc and Frc by invertases, *v*_Inv_. The free Hex can be phosphorylated by *v*_Glc_→_SP _and *v*_Frc_→_SP_, respectively. These reactions are catalysed by the enzymes glucokinase and fructokinase.

## Mathematical model structure

Time-dependent changes of metabolite concentrations during a diurnal cycle can be described by a system of ordinary differential equations (ODE). With **c **being the *m*-dimensional vector of metabolite concentrations, **N **being the *m *× *r *stoichiometric matrix and **v **being the *r*-dimensional vector of fluxes, the biochemical reaction network can be described as follows:(1)

with **v**(**c**,**p**) indicating that the fluxes are dependent on both, metabolite concentrations **c **and kinetic parameters **p**. Thus, based on the model structure (Figure 1) of our system, the concentration changes of the five-state variables: SP, St, sucrose, Glc and Frc are defined as:(2)

The stoichiometric coefficients account for the interconversions of species with a different number of carbon atoms. For example, the reaction *ν*_SP_→_Scr _has a stoichiometric coefficient value of 1 in the SP state equation, while in the Scr state equation, this value is 0.5 because SP contains 6 carbon atoms and Scr contains 12 carbon atoms. The stoichiometric coefficients for the reaction catalysed by invertase are 1 in all the respective state equations because this reaction represents the cleavage of the disaccharide Scr into two monosaccharides: Glc, and Frc. St content is expressed in Glc units, i.e. a carbohydrate with six carbon atoms. The rates of the ODE system (Equation 2) are determined in three ways: by measurements (model inputs), carbon balancing and kinetic rate laws.

## Model input and carbon balancing

The rate of net photosynthesis  was fed into the model using experimental data taken from [1]. Interpolated values of the measurements were applied to the SP state equation.

For modelling St synthesis and carbohydrate export, we used the following phenomenological approach. Although based on experimental data, the rate of net St synthesis was still subject to the identification process. It was defined as(3)

with *v*_St, min _and *v*_St, max _being derived from the measured concentration changes, i.e. the derivatives of the interpolated minimal and maximal concentrations. The parameter *p*_1 _varied between 0 and 1 and was determined in the process of parameter identification.

The rate of carbohydrate export(4)

was dynamically determined by balancing the external flux , the internal St flux *v*_St _and measured minimal and maximal total concentration changes of soluble carbohydrates *v*_C, min _and *v*_C, max_, respectively. *v*_C, min _and *v*_C, max _were calculated as already described for *v*_St, min _and *v*_St, max _by interpolating and differentiating with respect to time. In this way, the mechanistically and quantitatively unknown carbohydrate export can be calculated using measurement data of one flux  and two concentration changes (*v*_St_, *v*_C, min/max_). As with *p*_1_, the parameter *p*_2 _varied between 0 and 1 and was determined in the process of parameter identification.

This balancing formed the boundary condition for the system in Equation 2 and the model described the distribution of overall carbon flux through the internal reactions. The experimental setup as well as results of experimental data on carbohydrates and net photosynthesis are presented explicitly in [1].

## Kinetic rate equations

The rate of Scr synthesis (*v*_SP_→_Scr_) was assumed to follow a Michaelis-Menten enzyme kinetic:(5)

Rates of Scr cleavage (*v*_Inv_), Glc phosphorylation (*v*_Glc_→_SP_) and Frc phosphorylation (*v*_Frc_→_SP_) were defined by Michaelis-Menten kinetics including terms for product inhibition (Equations 6-8) as described in [3] and [4]:(6)(7)(8)

where *V*_max_(*t*) values represent time-variant maximal velocities of enzyme reactions, *K*_m _are the Michaelis-Menten constants representing substrate affinity of the enzyme and *K*_i _are the inhibitory constants. Changes in maximal velocities of enzyme reactions were described over a whole diurnal cycle by a cubic spline interpolation for *V*_max_(*t*). This course is defined by the sample *t*_*k *_= {3,7,11,15,19,23} h and values for *V*_max_(*t*_*k*_), which are subject to parameter identification. This description reflects changes of enzyme activity, mainly resulting from changes in enzyme concentration. Measurements of enzyme activities supported this assumption [1]. The kinetic rate law for the invertase reaction included a mixed inhibition by the products Glc and Frc, while hexose phosphorylation (*v*_Glc_→_SP_, *v*_Frc_→_SP_) was assumed to be inhibited non-competitively by SP. The model description, simulation and parameter identification was performed using the MATLAB SBToolbox2 [5].

## Parameter identification

Parameters were automatically adjusted applying a parameter-identification process representing the minimization of the sum of squared errors between measurement and simulation outputs by changing the parameter values within their bounds. For an overview of the formulation of such problems, see, e.g. [6]. In this context, the outputs which correspond to the model states are the concentration values of SP, St, Scr, Glc and Frc measured over a whole diurnal cycle at chosen time points. For a more detailed description of the quantification procedure and time points, refer to [1]. Measurements and simulations were carried out for *A. thaliana *wild type, accession Columbia (Col-0), and a knockout mutant *inv4 *defective in the dominating vacuolar invertase AtßFruct4 (At1G12240).

The final parameters have been identified using a particle swarm algorithm [7] that minimizes the sum of quadratic differences between measurement and simulation. This identification algorithm contains a stochastic component that enables overriding of local minima. We used the algorithm provided by the MATLAB/SBToolbox2 with its default options. The possible parameter ranges were constrained by different lower and upper bounds known from our own experiments (*V*_max_) and the literature (*K*_m_, *K*_i_). The model and the complete set of parameters and the best-fit comparison plots can be found in [1].

## Statistical fit analysis

**Figure 2:**
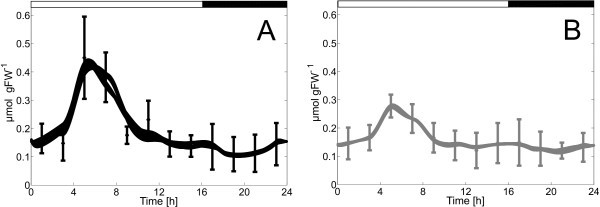
**Comparison of measurements (error bars: standard deviations; *N***_**r **_**= 5 replicates) and simulations (lines; *N***_**i,Col-0 **_**= 72 and *N***_**i,*inv4 ***_**= 71 identification runs) of Frc concentrations in leaf extracts**. **(a) **Wild-type (black), **(b) ***mutant *(grey). Time 0 h = 06:00 a.m. daytime. Concentrations are given in μmol per gFW (leaf fresh weight).

The model was intended not only to reproduce experimental data but also to allow predictions of variables and parameter values, for which no data were obtained. Therefore, the model was analysed for the variability of parameters and fluxes, which both are used for predictions. In [1], we performed 75 parameter-identification runs for the wild-type and the mutant. Within the chosen numerical accuracy, the algorithm converged to the same nominal cost function values in *N*_i,Col0 _= 72 and *N*_i,*inv4 *_= 71 cases, respectively. To give an impression of the fitting quality of the metabolite concentrations, all the *N*_i _simulation runs and measurements for both genotypes' Frc concentration are shown in Figure 2 exemplarily. The measurement error bars, i.e. the measurement standard deviations, are calculated from *N*_r _= 5 replications. The comparisons of measurements with simulations for the whole set of metabolites are shown in [1].

**Figure 3:**
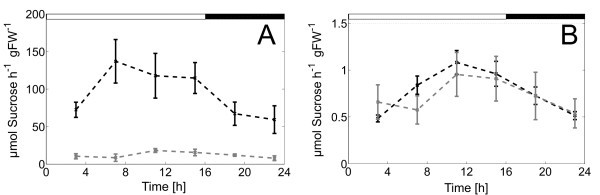
**Diurnal dynamics of (a) measured maximal invertase activity and (b) simulated rates of Scr cleavage (*v***_**Inv**_**) for wild-type (black lines) and *mutant *(grey lines)**. Values in **(a) **represent means ± SD (*N*_r _= 5 replicates), values in **(b) **represent means ± SD (identification runs: *N*_i,Col-0 _= 72, *N*_i,*inv4 *_= 71). Time 0 h = 06:00 a.m. daytime. Concentrations are given in μmol per gFW (leaf fresh weight).

We were able to identify significant differences in carbohydrate interconversion rates, which were not obvious and could not be determined by intuition [1]. For instance, one finding highlights the robustness of the considered system in spite of a significant reduction of the dominating activity of invertase in *inv4*. During the whole diurnal cycle, the calculated flux rates for the invertase reaction in wild-type and *inv4 *mutant differed considerably less than did the corresponding *V*_max _values for invertase (Figure 3). This observation indicated a possible stabilizing contribution of feedback mechanisms, for example, by product inhibition of invertase activity. In section "Stability properties of Scr cycling", this aspect is investigated further.

**Figure 4:**
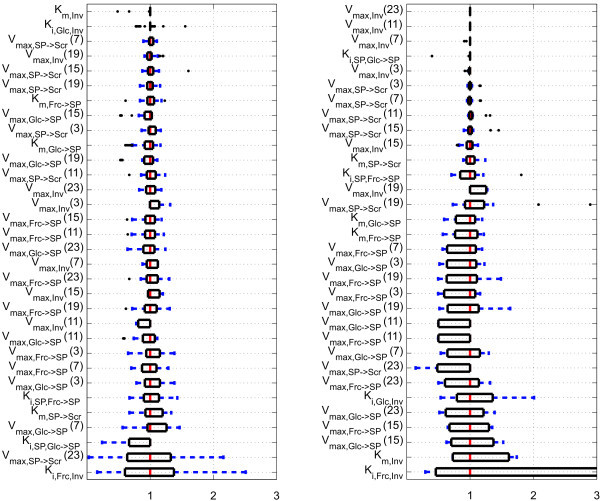
**Boxplots of identified kinetic parameters for wild-type (left side; *N***_**i,Col-0 **_**= 72) and mutant (right side; *N***_**i,*inv4 ***_**= 71)**. Numbers in brackets indicate time points (in hour) of time-variant parameters. Black dots represent outliers. The parameter *K*_i, Frc, Inv _of Col-0 has outliers at 21.7, 58.5 and 58.6. The upper quartile of the parameter *K*_i, Frc, Inv _of *inv4 *is at 37.6. *V*_max,SP_→_Scr _(23) of *inv4 *has outliers at 10.0.

**Figure 5:**
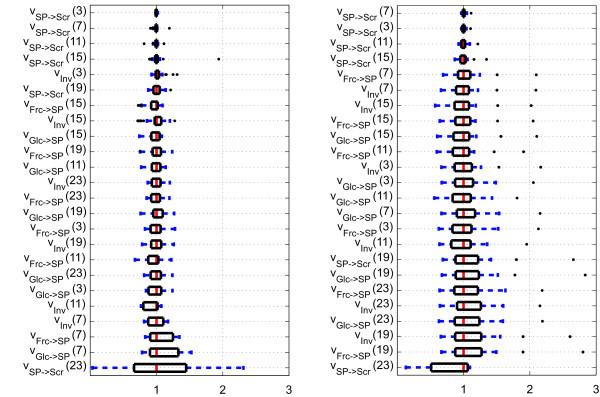
**Boxplots of the simulated metabolite fluxes for wild-type (left side; *N***_**i **_**= 72) and mutant (right side; *N***_**i **_**= 71)**. Numbers in brackets indicate time points in h. Black dots represent outliers. The flux *v*_SP_→_Scr_(23) of *inv4 *has outliers at 10.3 and 10.5.

Further, for displaying the variability of parameters, we chose boxplots that are superior in displaying distributions for skewed data sets, see, e.g. [8]. To compare identification results for different parameters, we scaled the identified values represented by their median and plotted distributions as *box-and-whisker *plots. The resulting graphs for all the parameters and flux values at the time points defined by the time-variant *V*_max _are shown in Figures 4 and 5. Outliers are displayed as dots. For a comparison of the parameter quality, values were sorted by their box width in the ascending order.

The parameter with the largest variability is the inhibition coefficient of fructokinase in both, the wild type and the mutant. Still, complete omission of inhibition structures leads to inferior simulation results (data not shown). Apart from the variability within the parameters, it can be observed that fluxes, such as *v*_*Inv*_, have smaller boxes than some of the associated kinetic parameters (here: *K*_i,Frc,Inv_), and that the wild type is less variable than the mutant (Figures 4 and 5). Further, the simulated concentrations show a relatively small variation (Figure 2). The result may be influenced by the number of runs, the algorithm's internal parameters, the algorithm itself or by the estimation bounds and should not be taken as confidence intervals of the parameter values. Therefore, the presented results only give an impression as to how the parameter variability is distributed for the chosen statistical setup.

Our observation that some parameter values have a much higher variability than the corresponding concentration and flux simulations is consistent with that of Gutenkunst et al. [9] in which many systems' biological models show the so-called sloppy parameter spectrum. Gutenkunst et al. [9] analysed several models with a nominal parameter vector **p**^o ^leading to nominal concentration courses. They studied the set of parameter values **p**, which lead to similar concentration courses as the nominal parameter values. For this purpose, they computed an ellipsoidal approximation of this set using the Hessian matrix of the χ^2 ^function, which is a measure for the deviation of the concentration courses from the nominal concentration courses. They found that in all the studied models, the lengths of the principal axes of this ellipsoid span several decades and are not aligned to the coordinate axes. Since parameters may vary along the long principal axes of the ellipsoid without significantly affecting the concentration courses, this means that many parameter values cannot be determined reliably by fitting the model to experimental data. At the same time, the model predictions may nevertheless be reliable.

We analysed whether an analogous property is found in our *N*_i _parameter sets. For this purpose, we performed a PCA [10]. PCA identifies the principal axes of a set of vectors. We applied a PCA to the set of vectors of logarithmic parameters that resulted from the convergent identification runs. For this purpose, we computed the covariance matrix **C **of the logarithmic parameter vectors such that *C*_*ij *_= cov(log(*p*_*i*_), log(*p*_*j*_)) corresponding to the *i*th and *j*th parameters, *p*_*i *_and *p*_*j*_, respectively. The eigenvectors of this matrix give the directions of the principal axes of the set of logarithmic parameter vectors. The eigenvalues correspond to the variances of the logarithmic parameters along the principal axes and present a measure for the lengths of the principal axes. An ellipsoid with these properties is given by Δ**p**^T^·**C**^-1^·Δ**p **≤ 1, where Δ**p **= log(**p**)-log(**p**°) is the deviation of the logarithmic parameter vector from its nominal value.

The longest principal axis of the mutant is approximately four times longer than the longest axis of the wild-type. This observation reflects the comparatively large boxes of the mutant box plots. For the mutant, the covariance matrix **C **is singular, with six eigenvalues being equal to zero within numerical tolerance. Two of those six eigenvalues correspond to the parameters describing the maximal velocity of the invertase reactions at two different time points (*V*_max,Inv _at *t *= 11 and 23 h) i.e. parameters directly connected to the mutation. These two parameters do not show a variation but are always at their bounds, which are much lower than in the wild-type. The analysis of the other four eigenvectors with eigenvalue zero revealed linear combinations of 29 parameters (all parameters except *V*_max,Inv_(11), *V*_max,Inv_(23) and *V*_max,SP_→_Scr_(23)), and their intuitive interpretation is not obvious.

**Figure 6:**
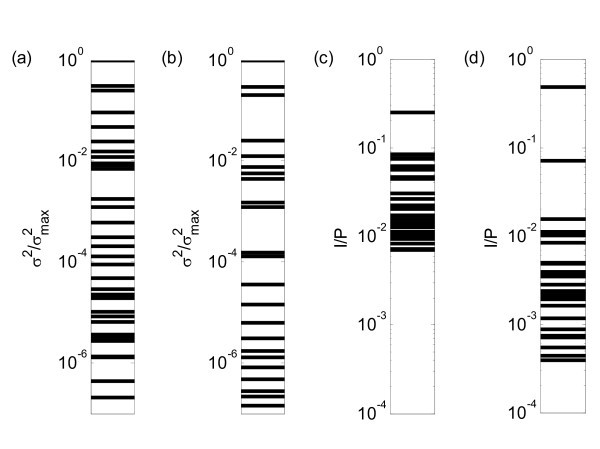
**Results of the principal component analysis**. Spectra of the principal components' variances (= eigenvalues of the covariance matrix) for wild-type **(a) **and *mutant ***(b)**. (Displayed values were scaled by the maximal variance. Some values are outside the displayed range). Spectra of the intersection/projection ratio (*I*/*P*) for wild type **(c) **and mutant **(d)**.

The above observations indicate that the parameter-identification problem for the mutant does not have a unique optimum, and the optima are on the border of the allowed area. For further analysis, we only analyse the principal axis with a non-zero variance. We removed six parameters from the parameter vector and computed the non-singular matrix **C **for the remaining parameters. The spectrum of the lengths of the principal axes is shown in Figure 6. The lengths were scaled such that the longest axis has a length of unity (10°). As expected for a sloppy system, the lengths of the principal axes span several orders of magnitude.

Next, we verified whether the principal axes are aligned with the coordinate axes. Gutenkunst et al. [9] suggest the use of the *I*_*i*_/*P*_*i *_ratio to quantify the alignment of the principal axes with the coordinate axes. Here, *I*_*i *_is the intersection of the ellipsoid with the *i*th coordinate axis and *P*_*i *_is the projection onto *i*th coordinate axis. A perfectly aligned principal axis has *I*_*i*_/*P*_*i *_= 1, whereas a skewed axis will lead to a deviation of unity. Gutenkunst et al. [9] give an expression to compute the *I*_*i*_/*P*_*i *_ratio on the basis of a quadratic form defining the ellipsoid. With our symbols, this expression is .

The *I*_*i*_/*P*_*i *_ratios span several orders of magnitude (Figure 6). This means that most principal axes are not aligned with the coordinate axes, as expected for a sloppy system.

In conclusion, the statistical analysis of the parameter vectors revealed three important properties of the system:

1. Different parameter-identification runs for the mutant converge to different edges of the allowed area. This fact reveals a problem with the identifiability of the model parameters for the mutant and explains the relatively large variation of the parameter values. In order to get a unique optimum, more experimental data of the previously unmeasured variables and a critical reassessment of the lower and upper bounds are needed.

2. The *N*_i _parameter sets show a sloppy parameter spectrum. This means that many parameter values cannot be reliably determined by parameter-identification algorithms that fit the model to experimental data.

3. The box plots in Figures 4 and 5 suggest which parameters and fluxes are likely to be determined reliably and which are not.

## Stability properties of Scr cycling

**Figure 7:**
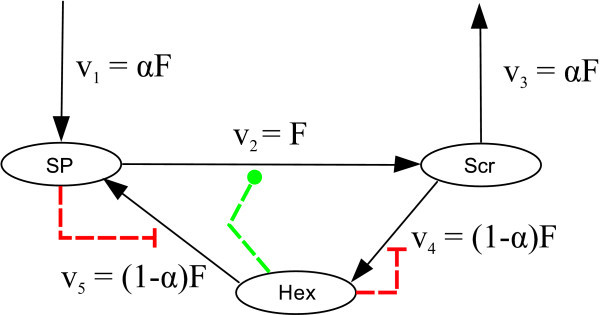
**Schematic representation of the metabolic cycle of Scr synthesis and degradation**. Inhibitory instances are indicated by red lines; activation is indicated by green lines. SP, sugar phosphates; Scr, sucrose; Hex, hexoses; *F*, reference flux; α, scaling parameter to describe fluxes as proportions of *F*.

As mentioned above, the knockout mutation of the dominant vacuolar invertase AtßFRUCT4 showed a dramatic reduction of cellular invertase activity, whereas the corresponding flux *v*_Inv _did not decrease in a corresponding manner (Figure 3). This finding indicated that the behaviour of the metabolic cycle of Scr degradation and re-synthesis are strongly determined by strong regulatory effects, as the product inhibition of invertase activity and of the synthesis of SP, as well as the activation of the synthesis of Scr by the Hex. Steady states in such strongly regulated systems are prone to instability, leading to effects as bi-stability or oscillations. The model defined by Equations 2, 5, 6, 7 and 8 approaches a stable steady state for given values of the in- and out-going reactions , *v*_St _and *v*_Scr_→_Sinks _if the overall carbon balance is fulfilled, i.e.  (data not shown). Diurnal dynamics are caused by the diurnal variations of these external fluxes and the diurnal changes of the enzyme activity. This means that we have a stable metabolic cycling whose diurnal dynamics are externally driven. In order to analyse the robustness of this scheme, we analysed the stability properties of the metabolic cycle by methods of structural kinetic modelling (SKM) as described in [11,12]. SKM is a specific application of generalized modelling [13] in which normalized parameters replace conventional parameters such as *V*_max _or *K*_m _in the modelling of metabolic networks. SKM in conjunction with a statistical analysis of the parameter space was used to determine whether a given steady state of a metabolite is always stable or whether it may be unstable for certain values of the normalized parameter [12]. We applied this methodology to our metabolic cycle of Scr degradation and synthesis, i.e. the central part of the system in consideration. In order to simplify the analysis, we summarised Glc and Frc as Hex. With this simplification, we obtained the network shown in Figure 7. Hex can activate *v*_2 _as described in [14]. Hex can also act as feedback inhibitors on v_4_, and v_5 _can be inhibited by the reaction product SP (Figure 7).

SKM allows analysing models with respect to given steady-state concentrations c_0_, and fluxes *v*_j_(c_0_). In this study, these values are subject to diurnal changes. However, the relative changes in concentration are small. Thus, we assumed steady-state concentrations of the metabolites, which we computed as the mean value of the concentrations over a whole day/night cycle. In steady state, flux *v*_1 _equals flux *v*_3 _= 6*v*_Scr_→_Sinks_. We set *v*_1 _= *v*_3 _= α*F*, where *F *represents the invertase flux. The parameter α can take values between 0 and 1 and determines the degree of Scr cycling. For α = 1, no cycling occurs. For α = 0, the cycling of carbon becomes maximal, and no carbon enters or leaves the cycle.

SKM defines normalised parameters with respect to the steady-state concentrations **c**_0 _and fluxes *v*_j_(c_0_):(9)(10)(11)

with *i *= 1...*m *(number of metabolites) and *j *= 1...*r *(number of reactions). The vector **x **describes the metabolite concentrations normalised based on their steady-state concentrations, the matrix **Λ **is the stoichiometric matrix normalised with respect to steady-state fluxes and steady-state metabolite concentrations, and **μ **represents the fluxes normalised related to steady-state flux values.

As described in [12], *x*_0 _= 1 represents the steady state of the system and the corresponding Jacobian *J *can be written as(12)

Each element of the matrix , analogue to scaled elasticities of metabolic control analysis, represents the degree of saturation of normalised flux μ_*j *_with respect to the normalised substrate concentration x_*i*_:(13)

thus indicating the degree of change in a flux as a particular metabolite is increased [11]. For irreversible Michaelis-Menten kinetics, as used in our kinetic model, the values in **θ **can assume values in the interval of [0,1]. In the case of allosteric inhibition by a product, as, for example, feedback inhibition of Hex on invertase enzymes, the corresponding element in **θ **assumes values within the range [-1,0]. Further details on **θ **for Michaelis-Menten kinetics can be found in [11]. The power of this approach lies in the ability to analyse the stability of the system by sampling combinations of the elements of **θ **which again represent combinations of the original kinetic parameters.

Considering the metabolic cycle shown in Figure 6 that contains three metabolites and five reactions, the following **Λ **(*m *× *r*) and **θ **(*r *× *m*) matrices can be developed:(14)(15)

**Figure 8:**
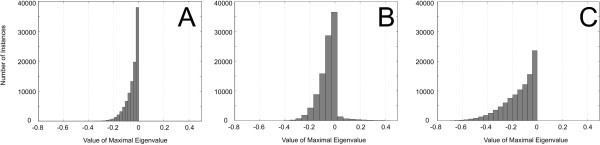
**Histograms of values of the maximal real part of eigenvalues for the metabolic system described in Figure 6**. **(a) **Histogram of the system without instances of activation or feedback inhibition; **(b) **histogram of the system with activation of *v*_2 _by Hex without feedback inhibition; and **(c) **histogram of the system with activation of *v*_2 _by HexHexHex and feedback inhibition of Hex on *v*_4 _and SP on *v*_5_.

**Figure 9:**
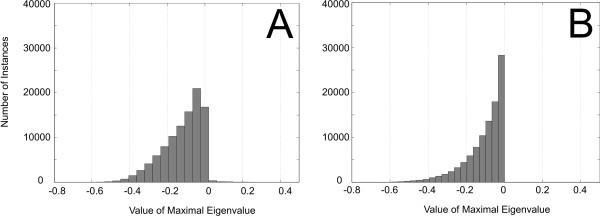
**Histograms of values of the maximal real part of eigenvalues for the metabolic system described in Figure 6**. **(a) **Histogram of the system with activation of *v*_2 _by Hex, weak feedback inhibition of SP on *v*_5 _and strong feedback inhibition of Hex on *v*_4_; **(b) **histogram of the system with activation of *v*_2 _by Hex, strong feedback inhibition of SP on *v*_5 _and weak feedback inhibition of Hex on *v*_4_.

The Jacobian matrix **J**_*x *_was calculated according to Equation 12. The system is guaranteed to be locally asymptotically stable if all eigenvalues of **J**_*x *_have negative real part. It is unstable, if one or more eigenvalues have positive real parts. The stability of nonlinear systems where all eigenvalues have non-positive real parts, but one which has a real part of zero, cannot be analysed with this approach. In the present setting, the latter case can be ignored since it occurs only for a lower dimensional subset of the parameter space. To explore stability properties of the considered Scr cycle, we performed computational experiments, in which the parameters in θ and α were set randomly following a standard uniform distribution on the open interval [0-1]. We analysed different modifications of the metabolic cycle by varying modes of activation and inhibition. Each particular metabolic cycle was simulated for 10^6 ^different sets of parameters, and resulting maximal real parts of the eigenvalues were plotted in histograms (Figures 8 and 9).

First, we analysed the stability properties of a system without instances of activation and inhibition (Figure 8a), i.e. by setting θ_2_, θ_5 _and θ_6 _to zero. All real parts of eigenvalues were negative, indicating stability for all the samples. Yet, if we considered *v*_2 _to be activated by Hex (θ_2 _> 0), positive real parts occurred, suggesting that the system may become unstable for certain parameter sets (Figure 8b). When additional instances of strong feedback inhibition (θ_5 _= θ_6 _= -0.99), e.g. by Hex or SP [1] were included, no positive eigenvalues appeared any more, and the system became stable again for all the tested parameter values (Figure 8c).

To determine whether feedback inhibition by Hex and SP contributed equally to stabilisation, we further analysed systems with (i) weak feedback inhibition of *v*_5 _by SP (θ_6 _= -0.01) and strong inhibition of *v*_4 _by Hex (θ_5 _= -0.99), and (ii) strong feedback inhibition of *v*_5 _by SP (θ_6 _= -0.99) and weak inhibition of *v*_4 _by Hex (θ_5 _= -0.01). The histograms representing the corresponding results showed that stability of the system for all the samples was only achieved when *v*_5 _was assumed to be inhibited strongly by SP (Figure 9a,b). Applying this theoretical model to a physiological context, reaction *v*_5 _would be represented by hexose phosphorylation through hexokinase enzymes, which have been shown to play a central role in sugar signalling, hormone signalling and plant development [15]. Our findings point to a strong influence of hexokinase on system stability and establishment of a metabolic homeostasis, supporting a crucial role in plant carbohydrate metabolism. In addition, a prevailing role of hexokinase in regulating Scr cycling would explain why a strong reduction of invertase activity caused only minor changes in the magnitude of Scr cycling in the *inv4 *mutant as already outlined in [1] (see Figure 3).

## Conclusions

Recently, we presented a kinetic modelling approach to simulate and analyse diurnal dynamics of carbohydrate metabolism in *A. thaliana*. Based on simulated fluxes in leaf cells, we could assign possible physiological functions of vacuolar invertase in carbohydrate metabolism. Here, we explicate this model in more detail and perform a statistical evaluation that proves reproducibility of the prediction of cellular metabolite concentrations and fluxes. The PCA revealed that the identifiability of the mutant parameters could be improved by more measurements. In addition, it was shown that this system's biology model exhibits the property of sloppiness [9], allowing for good predictions while some parameters show larger variability. The analysis of stability properties of Scr cycling indicated an important role of feedback inhibition mechanisms in stabilisation of futile metabolic cycles, and application of this concept to plant carbohydrate metabolism supported a role for hexokinase as a crucial regulator of Scr cycling.

## Competing interests

The authors declare that they have no competing interests.

## Abbreviations

Frc: fructose; Glc: glucose; Hex: hexoses; ODE: ordinary differential equations; PCA: principal component analysis; Scr: sucrose; SKM: structural kinetic modelling; SP: sugar phosphates; St: starch.
